# Effect of Sulfonamides and Their Structurally Related Derivatives on the Activity of *ι*-Carbonic Anhydrase from *Burkholderia territorii*

**DOI:** 10.3390/ijms22020571

**Published:** 2021-01-08

**Authors:** Viviana De Luca, Andrea Petreni, Alessio Nocentini, Andrea Scaloni, Claudiu T. Supuran, Clemente Capasso

**Affiliations:** 1 Institute of Biosciences and Bioresources, CNR, via Pietro Castellino 111, 80131 Napoli, Italy; viviana.deluca@ibbr.cnr.it; 2 Proteomics & Mass Spectrometry Laboratory, ISPAAM, CNR, via Argine 1085, 80147 Napoli, Italy; andrea.scaloni@ispaam.cnr.it; 3 Section of Pharmaceutical and Nutraceutical Sciences, Department of Neurofarba, University of Florence, via U. Schiff 6, 50019 Florence, Italy; andrea.petreni@unifi.it (A.P.); alessio.nocentini@unifi.it (A.N.)

**Keywords:** carbonic anhydrases, metalloenzyme, inhibitor, sulfonamides, kinetic constants

## Abstract

Carbonic anhydrases (CAs) are essential metalloenzymes in nature, catalyzing the carbon dioxide reversible hydration into bicarbonate and proton. In humans, breathing and many other critical physiological processes depend on this enzymatic activity. The CA superfamily function and inhibition in pathogenic bacteria has recently been the object of significant advances, being demonstrated to affect microbial survival/virulence. Targeting bacterial CAs may thus be a valid alternative to expand the pharmacological arsenal against the emergence of widespread antibiotic resistance. Here, we report an extensive study on the inhibition profile of the recently discovered ι-CA class present in some bacteria, including *Burkholderia territorii*, namely BteCAι, using substituted benzene-sulfonamides and clinically licensed sulfonamide-, sulfamate- and sulfamide-type drugs. The BteCAι inhibition profile showed: (i) several benzene-sulfonamides with an inhibition constant lower than 100 nM; (ii) a different behavior with respect to other α, β and γ-CAs; (iii) clinically used drugs having a micromolar affinity. This prototype study contributes to the initial recognition of compounds which efficiently and selectively inhibit a bacterial member of the ι-CA class, for which such a selective inhibition with respect to other protein isoforms present in the host is highly desired and may contribute to the development of novel antimicrobials.

## 1. Introduction

Carbonic anhydrases (CAs, EC 4.2.1.1) are essential metalloenzymes in nature, which speed up a fundamental reaction for all living organisms, the hydration of a molecule of carbon dioxide (CO_2_) into bicarbonate (HCO_3_^−^) and proton (H^+^), according to the following chemical reaction [1,2,3,4,5,6,7]: CO_2_ + H_2_O ⇋ HCO_3_^−^ + H^+^. The CA superfamily is grouped into eight CA classes indicated with the Greek letters (α, β, γ, δ, ζ, η, θ and ι), whose distribution is very variegated from the most complex organisms (plants and animals) to the simplest ones (bacteria and archaea) [1,2,3,4,5]. The genome of mammals, for example, encodes only for the α-CA class, of which 15 isoforms have been identified, which accomplish specialized functions in various tissues and organs [8,9,10,11,12]. Mammal breathing is a compelling example of the importance of these enzymes. In mammals, at the peripheral tissue level, the CO_2_ produced by aerobic metabolism leaves the cells and enters the bloodstream due to a pressure gradient effect [13]. About 90% of CO_2_ flows into red blood cells and is converted into bicarbonate by CAs. Then, the produced HCO_3_^−^ comes out from the red cell through an anion exchanger (AE) protein and is transported by the bloodstream to the lungs [13]. At the alveolar level, the concentration of CO_2_ is lower than in peripheral tissues, while there is a higher concentration of bicarbonate that is pumped into the red blood cell. Here, through the action of the inverse reaction catalyzed by CAs, the bicarbonate is transformed into water and CO_2_ [13]. The CO_2_ produced in this way is released into the bloodstream and, passing through the alveolus walls, is exhaled [13]. These reactions can also occur without the enzyme but carbonic anhydrase increases the reaction speed up to a million times (k_cat_ = 10^6^ s^−1^) [7]. At a physiological pH value, the spontaneous reversible CO_2_ hydration reaction in the absence of the catalyst has an effective first-order rate constant of 0.15 s^−1^, while the reverse reaction shows a rate constant of 50 s^−1^ [7]. Mammalian CAs are also involved in other important physiological processes, such as gluconeogenesis, lipogenesis and ureagenesis; transport of CO_2_/bicarbonate; electrolyte secretion in a variety of tissues/organs; bone resorption; calcification; renal and male reproductive tract acidification, signal transduction and formation of gastric acid [8,9,10,11,12].

In plants, α and β-CAs have an essential role in photosynthesis and biosynthetic reactions linked to it, in addition to few selected processes already mentioned above [14]. In simpler organisms, such as bacteria, Archaea and cyanobacteria α, β, γ and ι-CAs are present, with the function to balance the CO_2_/HCO_3_^−^ concentration ratio and a role in the carbon dioxide fixation [5,6,7,14,15,16]. Marine diatoms encode for α- 𝛿-, 𝜁-, θ- and 𝜄-CAs, which are involved in carbon dioxide fixation and metabolism [17,18,19]. In protozoa are present α-CAs and the recently discovered class, the η-CA, which is involved in *de novo* purine/pyrimidine biosynthetic pathways [20].

The significant progress made in the DNA sequencing approach allowed the identification of genes encoding for CAs in pathogenic and non-pathogenic microorganisms [21]. Intriguing, the understanding of the function of the bacterial CAs has increased significantly [6,22], confirming that the activity of CAs is connected to the survival as well as the virulence of pathogens because the metabolic reaction catalyzed by these enzymes is essential for supporting numerous physiological functions involving dissolved inorganic carbon [6,22]. Based on these considerations and in the time of emerging antibiotic resistance, targeting bacterial CAs towards a new generation of antibacterial drugs might represent a valid alternative to reinforce the pharmacological arsenal against these pathogens. Thus, scientists heterologously produced in vitro the CA-classes encoded by bacteria as well as other pathogens. Many data are available on kinetic parameters and inhibition profiles of the α-, β- and 𝛾-CAs encoded by pathogenic and non-pathogenic bacteria, while very few data are available on the latest discovered bacterial CA-class, the 𝜄-CA. This CA class was identified for the first time in the marine diatom *Thalassiosira pseudonana* [23] and, surprisingly, showed to prefer Mn^2+^ to Zn^2+^ as metal ion cofactor [23]. Generally, bacterial α- and β-CAs use the Zn^2+^ ion as catalytic metal, while γ-CAs are Fe^2+^-dependent enzymes but they are functional enzymes also with bound Zn^2+^ or Co^2+^ ions [24,25,26,27].

In a previous work, we have cloned, expressed and purified the first recombinant bacterial ι-CA (acronym BteCAι) identified in the genome of *Burkholderia territorii*, a Gram-negative bacterium found in soil and water, which is often resistant to common antibiotics [28,29]. The recombinant BteCAι was shown to be a suitable catalyst for the hydration of CO_2_ to bicarbonate and proton, with a k_cat_ of 3.0 × 10^5^ s^−1^ and k_cat_/K_M_ of 3.9 × 10^7^ M^−1^ s^−1^ and was also sensitive to inhibition by the sulfonamide acetazolamide [29]. Here, we carried out an extensive study on the inhibition profiles of BteCAι using the substituted benzene-sulfonamides and clinically licensed drugs, which, among the groups of the classical CAIs, generally inhibit other CAs in the nanomolar range and have been clinically used for decades as antiglaucoma [30], diuretic [15], antiepileptic [11], antiobesity [8,31] and anticancer agents [10]. Besides, a comparative analysis of the sulfonamide inhibition profiles was performed comparing BteCAι results with those obtained from CAs belonging to different classes, such as the two human α-CA isoforms (hCA I and hCA II) and the β-and γ-CAs from *Escherichia coli*. This study gives useful information for designing new antibacterials to disarm the pathogen or bypass their resistance to conventional antimicrobials by inhibiting enzymes involved in the CO_2_/HCO_3_^−^ balancing pathway.

## 2. Results and Discussion

### 2.1. Production, Validation and Qualitative Activity Assessment of BteCA𝜄

**Figure 1:**
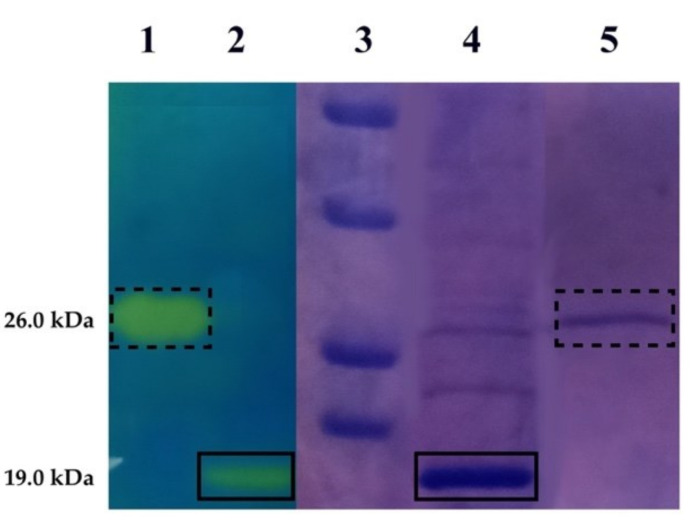
Combined lanes of sodium dodecyl-sulfate-polyacrylamide gel electrophoresis (SDS-PAGE) and protonography of BteCAι. Purified recombinant BteCAι (lane 4) was subjected to protonographic analysis (lane 2) to determine the enzyme activity on the polyacrylamide gel. Lane 3, molecular markers, from the top: 50.0 kDa, 37.0 kDa, 25 kDa and 20 kDa. Lane 1 and 5, commercial bovine carbonic anhydrase (bCA) used as control in SDS-PAGE (lane 5) and protonography (lane 1). Boxes with dashed and continuous lines indicate the bands identifying the bCA and BteCAι, respectively.

The ι-CA encoded by the genome of *B. territorii* was heterologously expressed in bacteria with the aim to produce a sufficient amount of protein for the determination of the corresponding inhibition profile with substituted benzene-sulfonamides and clinically licensed drugs. Sodium dodecyl-sulfate-polyacrylamide gel electrophoresis (SDS-PAGE) and protonography were used for the evaluation of the enzyme homogeneity and purity, as well as to verify the enzyme activity of BteCAι. As shown in Figure 1, SDS-PAGE analysis showed a protein migration corresponding to a molecular mass of 19.0 kDa. The BteCAι band was also active when subjected to protonography analysis, as demonstrated by the corresponding yellow color developed by the production of ions (H^+^) during the CO_2_ hydration reaction (Figure 1). Moreover, the protein concentration of the recombinant BteCAι was obtained from the SDS-PAGE, analyzing the gel by Image J and using a known concentration of the commercial bovine CA (bCA). Protein quantification by densitometry revealed that the E. coli cells overexpressed BteCAι with a yield of about 0.8 mg/mL from a bacterial culture of 1 L. The protein concentration calculated by densitometry resulted about 10% less with respect to that ascertained by the Bradford method. The BteCAι activity of the purified protein was also measured in solution and expressed in Wilbur-Anderson Units (WAU). BteCAι solution showed a specific activity of 80 ± 8.0 WAU mg^−1^.

### 2.2. Comparison of the BteCAι Kinetic Parameters with Those of Other Bacterial CA Classes

The CO_2_ hydratase activity and the kinetic constants of the purified BteCAι were determined using the stopped-flow technique. The enzyme had a high catalytic activity (k_cat_ 3.0 × 10^5^ s^−1^) for the physiological reaction of CO_2_ hydration to bicarbonate and protons. The enzyme was also well inhibited by the sulfonamide acetazolamide (K_I_ = 64.9 nM), a classical CA inhibitor. Table 1 shows a comparison of the BteCAι kinetic behavior (k_cat_ and k_cat_/K_M_) with those of other CAs belonging to different classes, such as two α-CA isoforms from Homo sapiens (hCAI and hCAII) and the β- and γ-CAs from E. coli.

**Figure 2:**
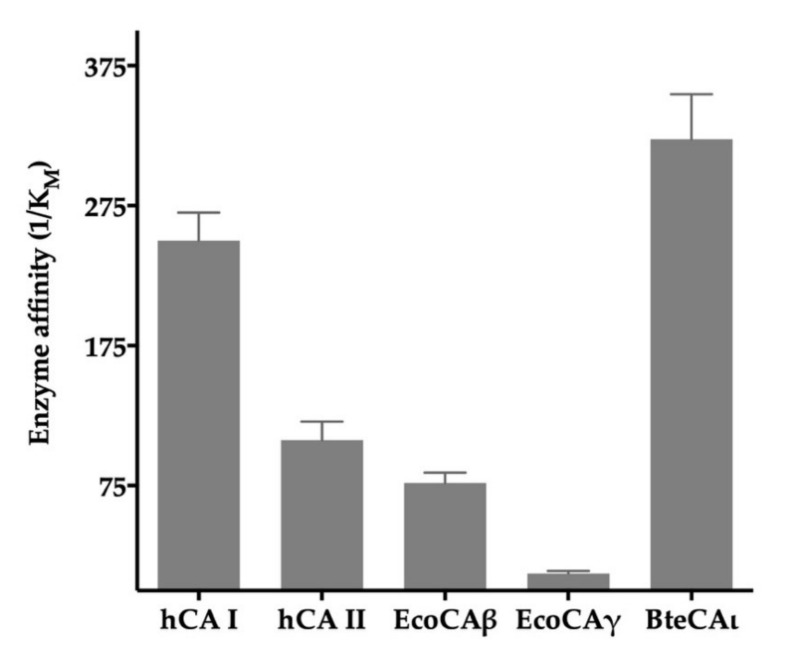
Graphical representation of the enzyme activity. The enzyme affinity for the CO_2_ was reported as 1/K_M_ to evidence that more remarkable is the bar height, more potent is the affinity of the enzyme for the substrate.

In Table 1, it is readily apparent that considered enzymes showed a catalytic constant (k_cat_, which indicates the maximum rate of the reaction at saturating substrate concentration) in the same order of magnitude, except for the human isoform CA II. Intriguing, the K_M_ value of BteCAι, the substrate concentration at which the reaction rate is half of V_max_, is one order of magnitude lower than that shown by the other two bacterial enzymes belonging to a different class (EcoCAβ and EcoCAγ). K_M_ is a measure of the enzyme’s affinity for its substrate and a lower K_M_ indicates that the enzyme accomplishes its function at a lower substrate concentration. Considering this aspect, the affinity for the substrate (CO_2_) of BteCAι is higher than that of the two human isoforms (hCA I and hCA II) as well as of the bacterial enzymes (β and γ). In particular, the affinity of BteCAι is 1.3- and 3.0-fold higher than that of hCA I and hCA II, respectively, whereas it is 4.2 to 26-fold higher compared with that of the other two bacterial enzymes (Figure 2).

In a previous work, we carried out a phylogenetic analysis to better evidence the amino acid sequence relationship of bacterial ι-CAs with other microorganism CA-classes (α, β and γ- CAs), demonstrating that the 𝜄-class is closer to the γ-CAs [29]. In the literature, it has been reported that ι-CA from the marine diatom *Thalassiosira pseudonana* shows a promiscuous esterase activity, which is a feature of the CAs belonging to the α-class [23]. Generally, the enzyme catalytic pocket is small for the γ-CAs and gets more ample for the β-CAs, being quite large in the α-CAs [6]. Based on these observations, it is intriguing to hypothesize that the ι-CAs have an active site spatial organization similar to that of the α-CA, for example, hCA I. However, the BteCAι spatial organization of the catalytic pocket probably allows a better entry of CO_2_ in the catalytic pocket as well as its tightly binding and thus a higher affinity for this molecule, as demonstrated by the low K_M_ value of BteCAι with respect to that of the human and the other two bacterial enzymes (β and γ-CAs) (Table 1 and Figure 2). Interestingly, although the ι-CA might have a catalytic pocket similar to the human CAs, the classical CA inhibitor, acetazolamide, inhibited BteCAι with a K_I_ of 519 nM, which is a value higher than those determined for other CAs considered in this study. All these findings suggest that even though these enzymes catalyze the same reaction, there are differences in the catalytic pocket, explaining the distinctive K_M_ and the diverse degree of inhibition shown by the various CA classes. Therefore, we are trying to crystallize the BteCAι with the aim to compare its three-dimensional structure with that reported in the literature for other known CAs. This analysis will allow the identification of the ι-CA structural elements responsible for such differences with respect to other CA classes.

### 2.3. Effects of Simple Aromatic/Heterocyclic Sulfonamide Inhibitors on BteCAι Activity

**Figure 3:**
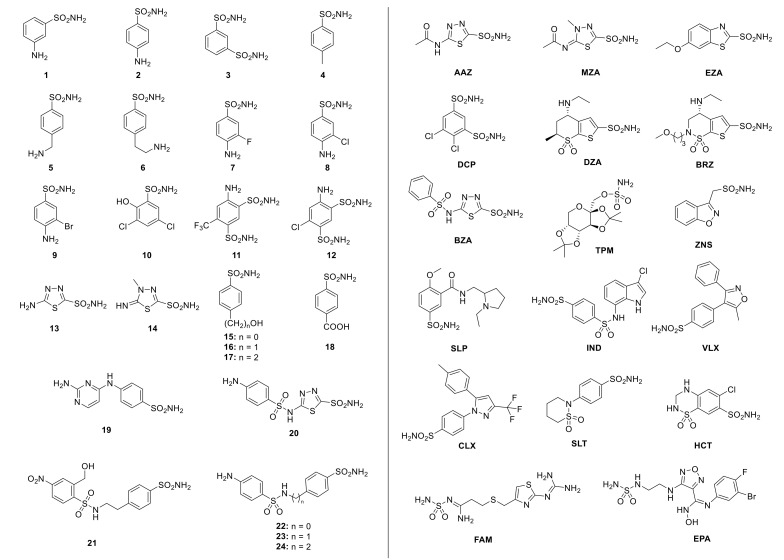
Sulfonamides and their structurally related derivatives, such as sulfamates and sulfamides, which have the general formula A-SO_2_NH_2_ (where A can be an aromatic, heterocyclic, aliphatic or sugar scaffold) and act as CAIs: simple aromatic/heterocyclic derivatives **1–24** (**left**); clinically used drugs or agents in clinical development (**right**).

The first sulfonamide showing a significant antibacterial activity was Prontosil, a sulfanilamide prodrug, which is isosteric/isostructural with *p*-aminobenzoic acid (PABA), the substrate of dihydropteroate synthase (DHPS) [32,33]. After sulfanilamide was demonstrated to be an effective antibacterial agent, a range of molecular analogs constituting the so-called sulfa drugs entered in clinical use [30]. The presence of primary sulfonamide moieties in sulfanilamide characterizes most investigated CA inhibitors (CAIs) [8,34,35,36,37]. Figure 3 shows some of these sulfonamide inhibitors (simple derivatives **1–24** and clinically used drugs) [3,38,39,40,41,42,43,44,45,46,47,48,49,50,51,52]. Acetazolamide (**AAZ**), methazolamide (**MZA**), ethoxzolamide (**EZA**) and dichlorphenamide (**DCP**) are systemically acting antiglaucoma CAIs. Dorzolamide (**DZA**) and brinzolamide (**BRZ**) are antiglaucoma agents that function topically; benzolamide (**BZA**) is an orphan drug of this pharmacological class. Zonisamide (**ZNS**), sulthiame (**SLT**) and topiramate (**TPM**) are widely used antiepileptic drugs. Sulpiride (**SLP**) and indisulam (**IND**) also belong to this class of pharmacological agents, together with the COX-2 selective inhibitors celecoxib (**CLX**) and valdecoxib (**VLX**). Saccharin (**SAC**) and the diuretic hydrochlorothiazide (**HCT**) are also known to act as CAIs [11,53,54]. Famotidine (**FAM**) is a competitive histamine H_2_-receptor antagonist [53] and epacadostat (**EPA**) is an inhibitor of the heme-containing enzyme, indoleamine 2,3-dioxygenase-1 (IDO1) but they also act as CAIs [54]. The aromatic/heterocyclic part of the inhibitor interacts with the hydrophilic and hydrophobic residues of the catalytic cavity. Its −SO_2_NH_2_ group binds in a tetrahedral geometry to the Zn^2+^ ion in the deprotonated state, with the nitrogen atom of the sulfonamide moiety coordinated to Zn^2+^ and an extended network of hydrogen bonds, involving amino acids of the enzyme, also participating in the anchoring of the inhibitor molecule to the metal ion, as shown by X-ray crystallographic data of enzyme-inhibitor adducts [11,53,54].

Numerous pieces of evidence support the involvement of CA activity in the survival, pathogenicity and virulence of several species of human pathogens, such as *Helicobacter pylori* [55,56,57], *Vibrio cholerae* [58], *Brucella suis* [52,59,60,61], *Salmonella enterica* [62] and *Pseudomonas aeruginosa* [63]. For example, ethoxzolamide (**EZA)** was demonstrated to inhibit the *V. cholerae* virulence in vivo by blocking the cholera toxin gene expression, induced by the bicarbonate produced by the Vibrio CA activity [58]; this inhibitor can also prejudice the virulence of *M. tuberculosis* [64]. Recently, it has been demonstrated that **AAZ** and some **AAZ**-based sulfonamides act as potent inhibitors of vancomycin-resistant *Enterococcus* [65], which are the leading causes of drug-resistant healthcare-associated infections. All these findings explain the proof of concept that bacterial CAs are promising targets for developing new drugs. Since CAs are valuable targets for compromising the microbial vitality or virulence, the BteCAι inhibition profile with these compounds can be considered an initial information for recognizing efficient and selective inhibitors of bacterial members of the ι-CA class in pathogens, with respect to other protein isoforms present in the host. This information is highly desired for obtaining new pharmacological agents, which may impair the bacterial growth/virulence. Table 2 compares the inhibition profiles of three classes of bacterial CAs and those obtained for the two human isoforms (hCA I and hCA II).

From the data of Table 2, the following results can be observed:

1. Among the sulfonamides and sulfamate used to determine the BteCAι inhibition profile, only three inhibitors resulted in a K_I_ lower than 100 nM. This is the case of the compounds **5**, **9** and **19**. These results confirm how different is the spatial organization of the catalytic pocket of the different CA classes. For the same compounds, the other two bacterial enzymes showed K_I_ values in the range 160–2840 nM, while the corresponding K_I_ values of two human isoforms were between 33–25,000 nM. The two human isoforms, hCA I and hCA II, resulted in a variegate behavior since hCA I was very susceptible to the inhibitors **9** and **19** with a K_I_ values of 40 and 33 nM, respectively. Again, both human isoforms showed many nanomolar inhibitors with a K_I_ below 100 nM, such as compounds **20**, **21**, **24**, **MZA**, **EZA**, **BZA**, **ZNS**, **IND**. Once more, there were compounds such as **3**, **7**, **10**, **11**, **12**, **13**, **14**, **15**, **16**, **DCP**, **DZA**, **BRZ**, **SLP**, **VLX** and **CLX**, which were potent inhibitors of hCA II with K_I_ values in the range 3–94 nM but were very week inhibitors of hCA I (K_IS_ > 5500 nM). Only the study of the three-dimensional structures of BteCAι (not available at this moment) will explain the structural factors responsible for the K_I_ variations.
2. Most of the inhibitors considered in Table 2 were moderate inhibitors of BteCAι with K_Is_ in the range 239–955 nM, such as compounds **1**, **2**, **3**, **4**, **6**, **7, 8, 10**, **11**, **12**, **13**, **14**, **15**, **16**, **17**, **20**, **21**, **22**, **23**, **24**, **AAZ**, **DCP**, **DZA**, **BZA**, **TMP**, **ZNS**, **SLP**, **SLT**, **IND**, **VLX**, **CLX**, **SLT**, **HTC**, **FAM** and **EPA**. A good number of these compounds, such as **1**, **2**, **3**, **14**, **20**, **21**, **AAZ**, **BZA** and **SLT,** resulted in moderate inhibitors for the other two bacterial enzymes (EcoCAβ and EcoCAγ), too. It is important to note that some of these inhibitors were very sensitive versus the human isoform hCA II but not versus the human isoform hCA I (K_IS_ > 10,000 nM). The zonisamide (**ZNS**), an aliphatic primary sulfonamide, was also a very weak inhibitor for the bacterial enzymes (K_Is_ = 755–3189 nM) but effective towards the human isoenzymes (K_Is_ = 35–56 nM).
3. Some substituted benzene-sulfonamides, such as **MZA**, **EZA**, **BRZ** and **SAC**, were rather ineffective, weak inhibitors of BteCAι, showing K_I_ values in the range of 5024–8466 nM. Moreover, **MZA** inhibited the *E. coli* EcoCAβ and EcoCAγ enzymes with K_I_ of 480 and 921 nM, respectively.

The results reported in Table 2 showed substantial differences in the inhibition constants between the various CA-classes. The inhibition pattern differences must be considered useful for the development of new specific drugs since it means that the spatiality and the amino acids surrounding the catalytic pocket of the CA enzymes are different. This diversity allows the possibility to design efficient and selective inhibitors of the bacterial enzymes without interfering with the activity of the human CAs, even if they show a high percentage of amino acid sequence identity.

## 3. Materials and Methods

### 3.1. Chemicals and Instruments

All the chemicals used in this study were of reagent grade and purchased from Sigma. The Affinity column (His-Trap FF) and the AKTA-Prime purification system were bought from GE Healthcare. SDS–PAGE apparatus was procured from Bio-Rad (Hercules, CA, USA).

### 3.2. Heterologous Expression and Purification of the Recombinant Enzyme

The synthetic *B. territorii* gene encoding for the BteCAι was cloned, expressed and purified, as described by Del Prete et al. [29]. Briefly, the synthetic gene contained in the expression vector pET100D-Topo/BteCAι was heterologously overexpressed, transforming the competent *E. coli* BL21 (DE3) codon plus cells (Agilent), using as Isopropyl β-D-1-thiogalactopyranoside (IPTG) as inducer. The produced cytoplasmic protein was purified by using a resin functionalized with Ni^2+^, which has a high affinity for the polyhistidine-tag (His-Tag) added by genetic engineering to the amino terminus of the neo-synthetized recombinant protein. The protein concentration was determined using the Bradford method (Bio-Rad) [66] and by densitometry using the Gel Analyzer tool of ImageJ [67].

### 3.3. Enzyme Activity, SDS-PAGE and Protonography

Throughout the purification, the proteolytic activity of BteCAι was achieved as described by Capasso et al. [68]. Twelve % SDS-PAGE performed as described by Laemmli [69] and protonography [70,71,72,73] carried out as reported by Del Prete et al. [29] were used to monitor the apparent molecular mass of the purified recombinant protein on the polyacrylamide gel and the corresponding hydratase activity, respectively.

### 3.4. Kinetic Parameters and Inhibition Constants

The CO_2_ hydration activity exerted by BteCAι was monitored using an Applied Photophysics stopped-flow instrument [74]. Phenol red (at a concentration of 0.2 mM) was used as an indicator, working at the absorbance maximum of 557 nm, with 20 mM TRIS (pH 8.3) as a buffer and 20 mM NaClO_4_ (for maintaining constant the ionic strength), following the initial rates of the CA-catalyzed CO_2_ hydration reaction for a period of 10–100 s. To determine the kinetic parameters by Lineweaver-Burk plots and the inhibition constants, a concentration of CO_2_ between 1.7 to 17 mM was used. For each inhibitor, at least three measurements were used to assess the initial velocity at all inhibitor concentrations tested. The uncatalyzed rates were identically determined and detracted from the total observed rates. Stock inhibitor solutions (10 mM) were prepared in distilled-deionized water and dilutions up to 0.1 nM were done with the buffer test. Inhibitor and enzyme solutions were preincubated together for 15 min, at room temperature, before the assay to allow for the formation of the E-I complex or the eventual active site mediated hydrolysis of the inhibitor. The inhibition constants were obtained by non-linear least-squares method using PRISM 6 and the Cheng-Prusoff equation, as reported earlier [75,76,77] and represent the mean from at least three different determinations. hCA I, hCA II and the bacterial enzymes were recombinant proteins obtained in-house. Their concentrations in the assay system were of 5–14 nM.

## 4. Conclusions

In this context, a broad range of substituted benzene-sulfonamides and clinically licensed drugs were used to determine the inhibition profile of BteCAι and the possible off-targets hCA I and hCA II. Among the sulfonamides and the one sulfamate used as inhibitors, only three of them resulted in having a K_I_ value lower than 100 nM (compounds **5**, **9** and **19**). All the other inhibitors had K_Is_ > 100 nM. Surprisingly, the results reported showed substantial differences in the inhibition constants between the various CA-classes considered in this study (α, β, γ and ι). For example, for some compounds EcoCAγ showed K_Is_ > 2000 nM, evidencing that this enzyme form or others *E. coli* froms were less or not inhibited by some of the substituted benzene-sulfonamides and clinically licensed drugs. These differences in the sulfonamide inhibition pattern represent an aspect of the CA inhibition useful for the development of specific and selective drugs versus the bacterial enzymes. It means that the spatiality and the amino acids surrounding the catalytic pocket of the CA enzymes are different. This diversity will allow the possibility to design inhibitors of the bacterial enzymes, which are efficient and selective versus the bacterial enzymes without interfering with the activity of human CAs, even if they show a significant percentage of amino acid sequence identity.

## Figures and Tables

**Table 1:** BteCAι kinetic parameters for the catalyzed CO_2_ hydration reaction and their comparison with those determined for different CA classes (α, β and γ). The kinetic measurements were carried out at 20 °C and pH 7.5 in 10 mM HEPES buffer for the hCA I, hCA II, EcoCA𝛾 and BteCAι, while a different buffer was used for the EcoCAβ enzyme (10 mM TRIS, pH 8.3, containing 20 mM NaClO4).

Organism	Enzyme Acronym	Class	k_cat_ (s^−1^)	K_M_ (M)	k_cat_/K_M_ (M^−1^·s^−1^)	K_I_ (Acetazolamide) (nM)
*Homo sapiens*	hCA I	α	2.0 × 10^5^	4.0 × 10^−3^	5.0 × 10^7^	250
	hCA II	α	1.4 × 10^6^	9.3 × 10^−3^	1.5 × 10^8^	12
*Escherichia coli*	EcoCAβ (CynT2)	β	5.3 × 10^5^	1.3 × 10^−2^	4.1 × 10^7^	227
	EcoCA𝛾	𝛾	5.7 × 10^5^	8.2 × 10^−2^	6.9 × 10^6^	248
*Burkholderia territorii*	BteCAι	𝜄	3.0 × 10^5^	3.1 × 10^−3^	9.7 × 10^7^	519

Mean from 3 different assays performed by a stopped flow technique (errors were in the range of ±5–10% of the reported values).

**Table 2:** BteCAι inhibition parameters for the catalyzed CO_2_ hydration reaction and their comparison with those determined for different CA classes (α, β and γ). The inhibition measurements were carried out at 20 °C and pH 7.5 in 10 mM HEPES buffer for the hCA I, hCA II, EcoCAγ and BteCAι, while a different buffer was used for the EcoCAβ enzyme (20 mM TRIS, pH 8.3, containing 20 mM NaClO_4_).

	K_I_ (nM) *				
Inhibitor	hCA I	hCA II	EcoCAβ	EcoCAγ	BteCAι
**1**	28,000	300	705	314	325
**2**	25,000	240	790	193	477
**3**	79,000	8	457	246	568
**4**	78,500	320	3015	221	446
**5**	25,000	170	2840	160	97
**6**	21,000	160	3321	622	786
**7**	8300	60	>10,000	605	481
**8**	9800	110	>10,000	671	346
**9**	6500	40	2712	718	96
**10**	7300	54	8561	2577	357
**11**	5800	63	6246	1779	239
**12**	8400	75	4385	1953	329
**13**	8600	60	4122	197	303
**14**	9300	19	440	712	434
**15**	5500	80	6445	1013	540
**16**	9500	94	2340	4238	594
**17**	21,000	125	502	1975	404
**18**	164	46	205	2064	467
**19**	109	33	416	1894	93
**20**	6	2	726	883	268
**21**	69	11	473	819	307
**22**	164	46	93	3501	365
**23**	109	33	322	4045	408
**24**	95	30	82	4262	698
**AAZ**	250	12	227	248	519
**MZA**	50	14	480	921	8466
**EZA**	25	8	557	5538	5024
**DCP**	1200	38	>10,000	889	825
**DZA**	50,000	9	629	2007	794
**BRZ**	45,000	3	2048	4842	3703
**BZA**	15	9	276	94	724
**TPM**	250	10	3359	648	787
**ZNS**	56	35	3189	755	806
**SLP**	1200	40	97	914	958
**IND**	31	15	2392	387	638
**VLX**	54,000	43	2752	891	934
**CLX**	50,000	21	1894	944	960
**SLT**	374	9	285	446	954
**SAC**	18,540	5959	6693	4903	7081
**HCT**	328	290	5010	3643	780
**FAM**	922	58	2769	274	943
**EPA**	8262	917	2560	744	955

* Mean from 3 different assays, by a stopped flow technique (errors were in the range of ± 5–10% of the reported values).

## Data Availability

The data presented in this study are openly available in https://pubmed.ncbi.nlm.nih.gov.
